# CT findings and features of postoperative abdominal infection patients with pancreatic carcinoma

**DOI:** 10.12669/pjms.333.12355

**Published:** 2017

**Authors:** Yuzhuo Ma, Guangsheng Liu, Lingling Zhang

**Affiliations:** 1 Yuzhuo Ma, Radiology Department, Binzhou People’s Hospital, Shandong 256610, China; 2 Guangsheng Liu Radiology Department, Binzhou People’s Hospital, Shandong 256610, China; 3 Lingling Zhang, Oncology Department, Binzhou People’s Hospital, Shandong 256610, China

**Keywords:** CT diagnosis, Pancreatic Carcinoma, Postoperative Abdominal Infection

## Abstract

**Objective:**

To investigate the values of Computed Tomography (CT) in diagnosing postoperative pancreatic surgeryabdominalinfection and its efficacy and to provide a reasonable method for the diagnosis of abdominal infection.

**Methods:**

Seventy-two patients who were confirmed as resectablepancreatic carcinoma by physical examination, CT, positron emission tomography (PET)/CT, endoscopic retrograde cholangiopancreatography (ER-CP), endoscopic ultrasonography and mesenteric angiography and were admitted to the Binzhou People’s Hospital, Shandong, China, from July 2013 to July 2015 were randomly selected. The plain CT images and clinical data of the patients were retrospectively analyzed.

**Results:**

Among 72 patients, 32 patients were diagnosed as abdominal infection by CT, three patients were misdiagnosed (two cases of intestinal obstruction and one case of intraperitoneal abscess), and 2 patients were wrongly diagnosed as suppurative abdominal inflammation. As regards distribution of CT imaging positive performance, the number of patients with intestinal loop abscess accounted for 41.7%, subphrenic abscess for 16.7%, pelvic abscess for 33.3%, the existence of septation for 25%, and emphysema sign for 16.7%. As to the distribution of CT findings of intestinal obstruction, 46.1% of patients had dilatation of intestine, 30.8% for bowel wall thickening, 7.7% had abnormal enhancement, 11.1% had density abnormality, and 15.4% had mesenteric effusion. CT features of purulent peritonitis showed 57.1% of patients had peritoneal thickening, 42.9% had peritoneal effusion, 42.9% had free intraperitoneal air, 14.3% had intestinal walls edema, and 28.6% had mesenteric edema.

**Conclusion:**

The diagnosis of postoperative abdominal infection of patients with pancreatic carcinoma using CT is quick and efficient showing the pattern and distribution of collection and the gross reaction to the exciting infection.

## INTRODUCTION

Pancreatic carcinoma is one of the commonly seen malignant tumors in the digestive system and also one of the malignant tumors with poor outcome. Pancreatic carcinoma can occurr in any site of pancreas, and pancreatic head has the highest incidence, with 60%~70%. Patients with pancreatic carcinoma show no obvious specific performance but clinical symptoms such as abdominal pain, jaundice, anorexia, body mass loss, nausea and emesis. The surgery associated mortality of pancreatic carcinoma patients is high; more than 90% of pancreatic carcinoma patients who have not underwent treatment died one year after definite diagnosis, and the average survival period is less than 6 months; therefore, the early pick up and diagnosis of pancreatic carcinoma is the key for improving curative effect.^1^-^4^ Currently, computed tomography (CT) is the major diagnostic method for pancreatic carcinoma. For patients with pancreatic carcinoma, suboptimal nursing can induce postoperative abdominal infection, which can further lower the survival rate of patients.^5^ The postoperative complications of patients with pancreatic carcinoma mainly include abscess shock and multiple organ failure, which can severely affect the quality of life of patients after surgery.^6^,^7^ Thus the accurate diagnosis of operation induced abdominal infection in the early stage is of great significance.

This study evaluated the values of CT in diagnosing postoperative pancreatic surgery abdominal infection and its efficacy and determined the infection frequency and CT patterns of different infections, aiming to provide a reasonable method for the diagnosis and treatment of abdominal infection.

## METHODS

Seventy-two patients who were diagnosed as pancreatic carcinoma by surgery and pathological slides in the Binzhou People’s Hospital from July 2013 to July 2015 were randomly selected. Their clinical performance mainly included upper abdominal pain, lacking in strength, jaundice and loss of appetite. Thirty-six patients had radiative pain on shoulder and back, 25 patients showed loss of appetite, and 11 patients were observed with mass in abdomen. All the patients had no organ infection, diabetes, hypertension and cardia-cerebrovascular disease before surgery. Moreover, they were confirmed as pancreatic carcinoma by physical examination, CT, positron emission tomography (PET)/CT, endoscopic retrograde cholangiopancreatography (ER-CP), endoscopic ultrasonography and mesenteric angiography. Patients successfully underwent laparoscopic resectionduring hospitalization, and their clinical data were preserved completely.

CT axial plain scan and enhancement scan were performed on all the cases using SOMATOM sensation 16 spiral CT machine (SIEMENS Inc., Germany). Scanning parameters were as follows: slice thickness: 5 mm, tube current: 320 mA, tube voltage: 120 KV, reconstruction slice thickness: two mm, reconstruction interval: two mm, scanning scope: from diaphragmatic dome to pubis. Enhancement scan including dual scan with 35 s and 70 s delay was carried out after the injection of contrast agent. According to the CT performance of patients and the clinical symptoms, relevant factors such as gastroenteric function were further analyzed. The incidence of abdominal opportunistic infection after surgery was recorded. The clinical data and various parameters of the patients before and after abdominal opportunistic infection were sorted.

### Statistical analysis

Data were processed and statistically analyzed using SPSS ver. 20.0. Difference was considered statistically significant if P<0.05.

## RESULTS

### CT diagnostic coincidence rate

Among 72 patients with pancreatic carcinoma, 32 patients were confirmed as abdominal infection by one1 case of intraperitoneal abscess; two cases were wrongly diagnosed as purulent peritonitis. The accuracy of the diagnosis was 84.4%.

### Abdominal infection after surgery

There were 13 cases of intestinal obstruction, 12 cases of intraperitoneal abscess and 7 cases of purulent peritonitis (40.6%, 37.5% and 21.9%).

### CT findings

The CT imagingof 13 cases of intestinal obstruction included dilatation of intestine, intestinal wall thickening and intestinal effusion; enhancement scan suggested abnormal enhancement of intestinal loop (Table-I). The CT findings of 12 cases of intraperitoneal abscess included low-density mass in peritoneal cavity, heterogeneous enhancement of irregular thick wall, and air fluid level which was seldom (Table-II). The major CTfindings of 7 cases of purulent peritonitis included ascites, peritoneal thickening, thickening and adhesion of intestinal wall, free intraperitoneal air and mesenteric edema (Table-III).

**Table-I:** Distribution of CT positive findings of patients with intestinal obstruction.

*CT findings*	*N*	*Proportion*
Dilatation of intestine	6	46.1%
Thickening of intestinal wall	4	30.8%
Abnormal enhancement	1	7.7%
Density abnormality	2	15.4%
Mesenteric effusion	2	15.4%

**Table-II:** Distribution of CT positive findings of patients with intraperitoneal abscess.

*CT findings*	*N*	*Proportion*
Intestinal loop abscess	5	41.7%
Subphrenic abscess	2	16.7%
Pelvic abscess	4	33.3%
Septation	3	25.0%
Emphysema sign	2	16.7%

**Table-III:** Distribution of CT findings of patients with purulent peritonitis.

*CT findings*	*N*	*Proportion*
Peritoneal thickening	4	57.1%
Peritoneal effusion	3	42.9%
Free intraperitoneal air	3	42.9%
Edema of intestinal wall	1	14.3%
Mesenteric edema	2	28.6%

## DISCUSSION

For patients with pancreatic carcinoma, suboptimal nursing can induce postoperative infection and various complications after surgery. The common complications which occur after pancreatic carcinoma surgery include pancreatic fistula, intestinal fistula, abdominal bleeding and infection, intraperitoneal septicemia and hepatic failure; some patients may have gastroperesis and septic shock after surgery.^8^ Difficult emission of fluid after surgery for intra-abdominal abscess can induce intolerance of cold, fever and intestinal peristalsis function disorder; delayed treatment may result in further invasion on blood vessels, intra-abdominal hemorrhage and intraperitoneal sepsis.^9^ The recurrence of pancreatic carcinoma is high after surgery; in most cases, the recurrence of pancreatic carcinoma occurs in the abdominal cavity. Therefore, the physical changes of patients should be carefully observed in the initial stage after surgery. Due to the weakening of body sensitivity after surgery, most patients cannot be aware of infection induced discomfort, leading to the delay of treatment. However, CT scan which can detect postoperative infected collections is beneficial for the diagnosis and managementof those complications.

Multi-slice spiral contrastenhanced scanning as a mature imaging examination method has been extensively used in examining the organs in the abdomen including the pancreas.^10^-^12^ Its importance lies in the diagnosis, identification, staging and resectability evaluation of pancreatic carcinoma have been proved by many studies.^13^ According to the patients’ evaluation after pancreatic carcinoma surgery, ultrasound is the preferred method; however it is limited in detecting retroperitoneal or gastrointestinal lesions. Therefore, CT is usually used for evaluating the abdominal symptoms of patients after pancreatic carcinoma surgery especially the patients with abdominal emergency or critically ill patients.^14^ The results of this study suggested that, 32 patients were diagnosed as abdominal infection by CT by the same attending doctor; three cases were missed, two2 cases of purulent peritonitis were misdiagnosed. The diagnostic accuracy was 84.4%, which was relatively satisfactory.

Abdominal infection after pancreatic carcinoma surgerymanifests as intraperitoneal abscess, intestinal obstruction and purulent peritonitis under CT.^15^ Intraperitoneal abscess should be distinguished with pancreatic pseudocyst, congenital mesenteric cyst and necrotic tumor. Even complete abscess wall enhancement and the existence of air signs are distinctive clues to the diagnosis of intraperitoneal abscess by CT. Purulent peritonitismanifests as thickened parietal peritoneum, seroperitoneum, pneumatosis, thickening and edema of omentum majus and mesentery of small intestine, etc. Those signs should be distinguished with tuberculous peritonitis which can also suggest symptoms of peritoneal thickening and ascites. But tuberculous peritonitis develops slowly and can be seen with tuberculous lesions in other organs.^16^

## CONCLUSION

In conclusion, abdominal infection has a high incidence after pancreatic carcinoma surgery and suggests multiple imaging manifestations. The diagnosis of abdominal infection after pancreatic carcinoma surgery by CT is of great clinical values to the early diagnosis of abdominal infectionassociated complications, beneficial to the improved control of complications, and can help the correct postoperative diagnosis and timely determination of treatment schemes.

### Authors’ Contribution

**YZM, GSL & LLZ:** Study design, data collection and analysis.

**YZM, GSL & LLZ:** Manuscript preparation, drafting and revising.

**GSL:** Review and final approval of manuscript.
